# Vital Signs: Alcohol Poisoning Deaths — United States, 2010–2012

**Published:** 2015-01-09

**Authors:** Dafna Kanny, Robert D. Brewer, Jessica B. Mesnick, Leonard J. Paulozzi, Timothy S. Naimi, Hua Lu

**Affiliations:** 1 Division of Population Health, National Center for Chronic Disease Prevention and Health Promotion, CDC; 2 Division of Unintentional Injury Prevention, National Center for Injury Prevention and Control, CDC; 3 Section of General Internal Medicine, Boston Medical Center, Boston, MA

## Abstract

**Background:**

Alcohol poisoning is typically caused by binge drinking at high intensity (i.e., consuming a very large amount of alcohol during an episode of binge drinking). Approximately 38 million U.S. adults report binge drinking an average of four times per month and consuming an average of eight drinks per episode.

**Methods:**

CDC analyzed data for 2010–2012 from the National Vital Statistics System to assess average annual alcohol poisoning deaths and death rates (ICD-10 codes X45 and Y15; underlying cause of death) in the United States among persons aged ≥15 years, by sex, age group, race/ethnicity, and state.

**Results:**

During 2010–2012, an annual average of 2,221 alcohol poisoning deaths (8.8 deaths per 1 million population) occurred among persons aged ≥15 years in the United States. Of those deaths, 1,681 (75.7%) involved adults aged 35–64 years, and 1,696 (76.4%) involved men. Although non-Hispanic whites accounted for the majority of alcohol poisoning deaths (67.5%; 1,500 deaths), the highest age-adjusted death rate was among American Indians/Alaska Natives (49.1 per 1 million). The age-adjusted rate of alcohol poisoning deaths in states ranged from 5.3 per 1 million in Alabama to 46.5 per 1 million in Alaska.

**Conclusions:**

On average, six persons, mostly adult men, die from alcohol poisoning each day in the United States. Alcohol poisoning death rates vary substantially by state.

**Implications for Public Health Practice:**

Evidence-based strategies for preventing excessive drinking (e.g., regulating alcohol outlet density and preventing illegal alcohol sales in retail settings) could reduce alcohol poisoning deaths by reducing the prevalence, frequency, and intensity of binge drinking.

## Introduction

Excessive alcohol use accounted for an average of one in 10 deaths among working-age adults (aged 20–64 years) in the United States each year during 2006–2010 (1), and cost the United States $223.5 billion in 2006 (2). Binge drinking, defined as consuming four or more drinks for women or five or more drinks for men on an occasion, was responsible for more than half of these deaths (1) and three fourths of the economic costs (2). Binge drinking also is responsible for many health and social problems, including alcohol poisoning (3). Yet, approximately 38 million U.S. adults report binge drinking an average of four times per month, and consume an average of eight drinks per binge episode (4). Most binge drinkers (90%) are not alcohol dependent (5).

Alcohol poisoning is typically caused by binge drinking at high intensity. Such drinking can exceed the body’s physiologic capacity to process alcohol, causing the blood alcohol concentration to rise. The clinical signs and symptoms of alcohol intoxication are progressive, and range from minimal impairment, decreased judgment and control, slurred speech, reduced muscle coordination, vomiting, and stupor (reduced level of consciousness and cognitive function) to coma and death. However, an individual’s response to alcohol is variable depending on many factors, including the amount and rate of alcohol consumption, health status, consumption of other drugs, and metabolic and functional tolerance of the drinker (6,7).

Reducing the proportion of adults engaging in binge drinking (objective SA-14.3) and reducing the number of deaths attributable to alcohol (objective SA-20), including deaths from alcohol poisoning, are among the objectives in *Healthy People 2020* (8). Reducing drug abuse and excessive alcohol use are also key components of the National Prevention Strategy (9).

## Methods

CDC analyzed multiple cause-of-death mortality files for 2010–2012 from the National Vital Statistics System (10) to assess average annual alcohol poisoning deaths among persons aged ≥15 years in the United States. Alcohol poisoning deaths were defined as those with *International Classification of Diseases, 10th Revision* (ICD-10) underlying (i.e., principal) cause of death codes X45 (accidental poisoning by and exposure to alcohol) and Y15 (poisoning by and exposure to alcohol, undetermined intent). Alcohol poisoning death rates per 1 million were calculated by sex, age group, and race/ethnicity for persons aged ≥15 years using the U.S. Census bridged-race population for 2010–2012 as the denominator, and were age-adjusted to the 2000 U.S. Census standard population. State death rates also were calculated and age-adjusted to the 2000 U.S. Census standard population.

**Key Points**

- An annual average of 2,221 alcohol poisoning deaths, or six deaths per day, occurred in the United States during 2010–2012.
- Alcohol poisoning is typically caused by binge drinking at high intensity (i.e., consuming a very large amount of alcohol during an episode of binge drinking).
- Three in four of those who died were adults aged 35–64 years, and three in four decedents were men.
- Almost 70% of the deaths were among non-Hispanic whites; however, the highest age-adjusted alcohol poisoning death rate was among American Indians/Alaska Natives (49.1 deaths per 1 million).
- The age-adjusted alcohol poisoning death rate in states ranged from 5.3 deaths per 1 million in Alabama to 46.5 deaths per 1 million in Alaska.
- Several evidence-based strategies effective in reducing excessive alcohol use and related harms have been identified and recommended.
- Additional information is available at http://www.cdc.gov/vitalsigns.

Selected conditions that might have directly contributed to alcohol poisoning deaths, including alcohol dependence (F10.2), hypothermia (X31, T68, T69.9), drug poisoning (T36–T50), and drug use mental disorders (F11–F16, F18, F19), also were assessed among persons who died of alcohol poisoning.

## Results

During 2010–2012, there was an annual average of 2,221 alcohol poisoning deaths, an age-adjusted rate of 8.8 deaths per 1 million population, among persons aged ≥15 years in the United States (Table 1). Of these deaths, 1,681 (75.7%) were among adults aged 35–64 years, and 1,696 (76.4%) were among men. The highest death rate from alcohol poisoning was among men aged 45–54 years (25.6 deaths per 1 million). Although non-Hispanic whites accounted for the majority of alcohol poisoning deaths (67.5%; 1,500 deaths), the highest age-adjusted alcohol poisoning death rate was among American Indians/Alaska Natives (49.1 deaths per 1 million). A total annual average of 44 deaths (2.0%) involved persons aged 15–20 years, who were under the legal drinking age of 21.

The age-adjusted alcohol poisoning death rate in states ranged from 5.3 per 1 million in Alabama to 46.5 per 1 million in Alaska (Table 2). Twenty states had alcohol poisoning death rates greater than the overall national rate of 8.8 per 1 million, and two states (Alaska and New Mexico) had alcohol poisoning death rates >30 per 1 million. States with the highest death rates were located mostly in the Great Plains and western United States, but also included two New England states (Rhode Island and Massachusetts) (Figure).

Alcohol dependence was listed as a contributing cause of death in an annual average of 677 (30.4%) of the deaths from alcohol poisoning, and hypothermia was listed as a contributing cause of death in an annual average of 134 (6.0%) deaths. Drug poisoning and drug use mental disorders were listed as contributing causes of death in an annual average of 62 (2.8%) and 86 (3.9%) deaths from alcohol poisoning, respectively.

## Conclusions and Comment

The results in this report indicate that during 2010–2012 there was an average of six deaths from alcohol poisoning each day among persons aged ≥15 years in the United States. Three in four of these deaths involved adults aged 35–64 years, and three in four of these deaths involved males. Nearly 70% of the deaths were among non-Hispanic whites; however, the highest alcohol poisoning death rate was among American Indians/Alaska Natives (49.1 deaths per 1 million).

The large proportion of alcohol poisoning deaths (75.7%) among adults aged 35–64 years is consistent with recent findings that two thirds (69%) of all average annual alcohol-attributable deaths in the United States involve adults aged 20–64 years (1). Alcohol-attributable deaths also result in substantial losses in workplace productivity and were responsible for >70% of the $223.5 billion in economic costs attributed to excessive drinking in the United States in 2006 (2). This finding also is consistent with the distribution of binge drinking episodes in the United States, most of which are reported by adults aged ≥26 years (11).

The large proportion of alcohol poisoning deaths among non-Hispanic whites is consistent with the high prevalence of binge drinking in this population (4). The high alcohol poisoning death rate among American Indians/Alaska Natives also is consistent with the high binge drinking intensity that has been reported by binge drinkers in this population (4). A recent study found that American Indians/Alaska Natives were seven times more likely to die from alcohol poisoning than whites, reflecting both the higher intensity of binge drinking among binge drinkers in this population and other factors, such as geographic isolation and reduced access to medical care (12).

Differences in alcohol poisoning death rates in states reflect known differences in state binge drinking patterns, which are strongly influenced by state and local laws governing the price and availability of alcohol (13), as well as other cultural and religious factors (14). A recent study that examined the relationship between various subgroups of state alcohol policies and binge drinking among adults found that a small number of policies that raised alcohol prices and reduced its availability had the greatest impact on binge drinking in states (15). However, other factors, in addition to differences in binge drinking rates, also might be important contributors to differences in alcohol poisoning death rates. For example, living in geographically isolated rural areas might increase the likelihood that a person with alcohol poisoning will not be found before death or that timely emergency medical services will not be available.

Although alcohol dependence was a contributing cause of death in 30% of alcohol poisoning deaths, the majority of these deaths involved persons for whom alcohol dependence was not listed as a contributing cause of death. This result is consistent with the results of a recent study that found that nine in 10 adults who drink excessively were not alcohol dependent, including more than two thirds of those who reported binge drinking ≥10 times per month (5).

The findings in this analysis are subject to at least three limitations. First, alcohol-attributable deaths, including alcohol poisoning, are underreported (16–18). Second, this study was restricted to deaths in which alcohol poisoning was the underlying cause of death, and did not include deaths in which alcohol poisoning was a contributing cause of death. A previous study found that there were three times as many deaths in which alcohol poisoning was a contributing, rather than underlying cause of death (19). Finally, mortality data might underestimate the actual number of deaths for American Indians/Alaska Natives (12) and certain other racial/ethnic populations (e.g., Hispanics) because of misclassification of race/ethnicity of the decedents on death certificates (20).

There are several recommended evidence-based, population-level strategies to reduce excessive drinking and related harms, such as regulating alcohol outlet density (i.e., the concentration of retail alcohol establishments, including bars and restaurants and liquor or package stores, in a given geographic area) and preventing illegal alcohol sales in retail settings (e.g., commercial host [dram shop] liability) (21,22). The status of each state’s policies related to some of these recommendations are available from CDC online (at http://www.cdc.gov/psr/alcohol). Screening and brief intervention for excessive alcohol use, including binge drinking, among adults has also been recommended (23). However, a recent study found that only one in six U.S. adults overall, one in five current drinkers, and one in four binge drinkers in 44 states and the District of Columbia reported ever discussing alcohol use with a doctor or other health professional. Furthermore, 65.1% of those who reported binge drinking ≥10 times in the past month had never had this dialogue (24).

Death from alcohol poisoning is a serious and preventable public health problem in the United States. A comprehensive approach to the prevention of excessive drinking that includes evidence-based community and clinical prevention strategies is needed to decrease alcohol poisoning deaths and other harms attributable to excessive alcohol use.

## Figures and Tables

**FIGURE:**
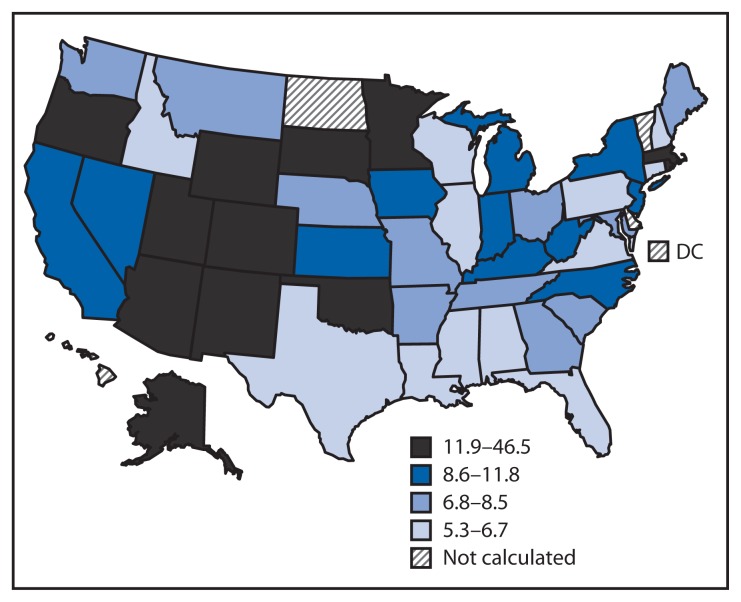
Age-adjusted alcohol poisoning* death rates,^†^ by state^§^ — National Vital Statistics System, United States, 2010–2012 * Alcohol poisoning deaths included those occurring among those aged ≥15 years in which alcohol poisoning was classified as the underlying (i.e., principal) cause of death based on *International Classification of Diseases, 10th Revision* (ICD-10) codes X45 (accidental poisoning by and exposure to alcohol) and Y15 (poisoning by and exposure to alcohol, undetermined intent). ^†^ Rates per 1 million population for persons aged ≥15 years were calculated using U.S. Census bridged-race population for 2010–2012, and were age-adjusted to the 2000 U.S. Census standard population. ^§^ The average annual number of alcohol poisoning deaths in Delaware, District of Columbia, Hawaii, North Dakota, and Vermont was less than seven and therefore, did not meet standards of reliability and precision to calculate age-adjusted death rates.

**TABLE 1:** Alcohol poisoning deaths,* by sex, age group, and race/ethnicity — National Vital Statistics System, United States, 2010–2012

Characteristic	Total			Male			Female		
		
	Average annual no. of deaths	% of total deaths	Age-adjusted rate†	Average annual no. of deaths	% of male deaths	Age-adjusted rate†	Average annual no. of deaths	% of female deaths	Age-adjusted rate†
**Overall**	**2,221**	**100.0**	**8.8**	**1,696**	**100.0**	**13.7**	**525**	**100.0**	**4.1**
**Age group**§ **(yrs)**									
15–24	113	5.1	2.6	85	5.0	3.8	28	5.4	1.3
25–34	288	13.0	6.9	228	13.4	10.9	60	11.4	2.9
35–44	476	21.4	11.7	370	21.8	18.2	106	20.2	5.2
45–54	747	33.6	16.7	564	33.3	25.6	183	34.8	8.1
55–64	458	20.6	12.2	352	20.7	19.3	107	20.3	5.5
≥65	139	6.3	3.3	98	5.8	5.4	41	7.9	1.8
**Race/Ethnicity**									
White, non-Hispanic	1,500	67.5	8.8	1,103	65.0	13.1	397	75.6	4.6
Black, non-Hispanic	191	8.6	6.2	149	8.8	10.6	42	8.1	2.6
Hispanic	338	15.2	9.0	296	17.5	15.6	41	7.9	2.4
American Indian/Alaska Native	154	6.9	49.1	114	6.7	75.0	39	7.5	24.3
Asian/Pacific Islander	32	1.5	2.2	28	1.7	4.1	4	0.8	—¶

* Alcohol poisoning deaths included those occurring among persons aged ≥15 years in which alcohol poisoning was classified as the underlying (i.e., principal) cause of death based on *International Classification of Diseases, 10th Revision* (ICD-10) codes X45 (accidental poisoning by and exposure to alcohol) and Y15 (poisoning by and exposure to alcohol, undetermined intent).

† Rates per 1 million population for persons aged ≥15 years were calculated using U.S. Census bridged-race population for 2010–2012, and were age-adjusted to the 2000 U.S. Census standard population.

§ Age-specific rate.

¶ Number of deaths was too small to meet standards of reliability and precision to calculate age-adjusted death rate.

**TABLE 2:** Average annual number of alcohol poisoning deaths,* by state — National Vital Statistics System, United States, 2010–2012

State†	Average annual no. of deaths	Age-adjusted rate§
**Quartile 1 (5.3–6.7 death rate)**		
Alabama	20	5.3
Texas	109	5.4
Illinois	57	5.6
Virginia	40	5.9
Wisconsin	28	6.0
Idaho	8	6.1
Louisiana	22	6.2
Pennsylvania	68	6.5
Connecticut	19	6.6
Florida	103	6.7
Mississippi	15	6.7
New Hampshire	8	6.7
**Quartile 2 (6.8–8.5 death rate)**		
Ohio	64	6.9
South Carolina	28	7.4
Missouri	38	7.7
Tennessee	41	7.8
Georgia	62	7.8
Arkansas	17	7.8
Maryland	37	7.8
Washington	46	8.1
Maine	9	8.1
Nebraska	11	8.1
Montana	7	8.5
**Quartile 3 (8.6–11.8 death rate)**		
Indiana	43	8.6
North Carolina	68	8.6
New York	143	8.8
Kentucky	32	9.1
Kansas	22	9.6
Iowa	23	9.7
Michigan	77	9.7
Nevada	21	9.8
New Jersey	74	9.9
California	299	9.9
West Virginia	17	11.2
**Quartile 4 (11.9–46.5 death rate)**		
Massachusetts	67	11.9
Oklahoma	37	12.6
Oregon	42	12.7
Colorado	60	14.4
Minnesota	73	16.4
Utah	33	16.7
South Dakota	11	17.0
Wyoming	8	17.7
Arizona	93	18.7
Rhode Island	21	22.8
New Mexico	52	32.7
Alaska	27	46.5

* Alcohol poisoning deaths included those occurring among those aged ≥15 years in which alcohol poisoning was classified as the underlying (i.e., principal) cause of death based on *International Classification of Diseases, 10th Revision* (ICD-10) Codes: X45 (Accidental poisoning by and exposure to alcohol), Y15 (Poisoning by and exposure to alcohol, undetermined intent).

† The average annual number of alcohol poisoning deaths in Delaware, District of Columbia, Hawaii, North Dakota, and Vermont was less than seven and therefore, did not meet standards of reliability and precision to calculate age-adjusted death rates.

§ Rates per 1 million population for persons aged ≥15 years were calculated using U.S. Census bridged-race population for 2010–2012, and were age-adjusted to the 2000 U.S. Census standard population.
